# Multiparametric Microstructural MRI and Machine Learning Classification Yields High Diagnostic Accuracy in Amyotrophic Lateral Sclerosis: Proof of Concept

**DOI:** 10.3389/fneur.2021.745475

**Published:** 2021-11-17

**Authors:** Thomas D. Kocar, Anna Behler, Albert C. Ludolph, Hans-Peter Müller, Jan Kassubek

**Affiliations:** ^1^Department of Neurology, University of Ulm, Ulm, Germany; ^2^German Center for Neurodegenerative Diseases (DZNE), Ulm, Germany

**Keywords:** diffusion tensor imaging (DTI), machine learning, support vector machine (SVM), neural network, amyotrophic lateral sclerosis, motor neuron disease, neurodegeneration, magnetic resonance imaging (MRI)

## Abstract

The potential of multiparametric quantitative neuroimaging has been extensively discussed as a diagnostic tool in amyotrophic lateral sclerosis (ALS). In the past, the integration of multimodal, quantitative data into a useful diagnostic classifier was a major challenge. With recent advances in the field, machine learning in a data driven approach is a potential solution: neuroimaging biomarkers in ALS are mainly observed in the cerebral microstructure, with diffusion tensor imaging (DTI) and texture analysis as promising approaches. We set out to combine these neuroimaging markers as age-corrected features in a machine learning model with a cohort of 502 subjects, divided into 404 patients with ALS and 98 healthy controls. We calculated a linear support vector classifier (SVC) which is a very robust model and then verified the results with a multilayer perceptron (MLP)/neural network. Both classifiers were able to separate ALS patients from controls with receiver operating characteristic (ROC) curves showing an area under the curve (AUC) of 0.87–0.88 (“good”) for the SVC and 0.88–0.91 (“good” to “excellent”) for the MLP. Among the coefficients of the SVC, texture data contributed the most to a correct classification. We consider these results as a proof of concept that demonstrated the power of machine learning in the application of multiparametric quantitative neuroimaging data to ALS.

## Introduction

Amyotrophic lateral sclerosis (ALS) is a clinically and genetically heterogeneous, multidomain neurodegenerative syndrome of motor and extra-motor systems with multiple different clinical subphenotypes and alterations in several brain regions, most prominently the corticospinal tract (CST) (1). The potential of multiparametric, quantitative magnetic resonance imaging (MRI) in the diagnostic procedures of ALS is widely recognized (2, 3). The development of current machine learning algorithms introduced the opportunity to combine biomarkers from different MRI metrics into a single classifier, even in more complex settings. Previous approaches using resting state functional MRI (rs-fMRI), T1 weighted imaging (T1w), and diffusion tensor imaging (DTI) data have achieved diagnostic accuracy of well over 70% (4–6), recently summarized in a systematic review (7). The challenge in designing a suitable machine learning model remains the appropriate selection of features and the collection of a meaningful amount of data to build a reliable model (8). In addition to traditional techniques, texture analysis is among the most promising approaches for disease classification in ALS (9, 10). The combination of texture analysis with traditional diffusion metrics makes a multiparametric microstructural assessment possible that might enhance diagnostic accuracy in machine learning classifiers. In the present study, a retrospective data analysis with T1w and DTI data was conducted. To this end, we extracted diffusion metrics of the most important tracts in ALS as well as texture data from the motor segment of the corpus callosum. We combined these data in a linear support vector classifier (SVC) which is a robust model that can give feedback on feature importance, with the prospect of providing a proof of concept model with high accuracy and unraveling underlying patterns that could help to build future classifiers. Furthermore, we used a state-of-the-art artificial neural network to verify the results. As a proof of concept study, we primarily focused on the general feasibility of the approach, not the optimization of the ML algorithm.

## Materials and Methods

### Data Collection and Preprocessing

The data were collected from the MRI data archive of the Dept of Neurology, University of Ulm, Germany and included 1.5 T imaging data sets that contained a high-resolution T1w sequence and also a DTI sequence with at least 39 gradients. The search resulted in 404 data sets from ALS patients (237 male; mean age 63 ± 11 years) and 98 data sets from healthy control subjects (49 male; mean age 57 ± 16 years), recorded between May 2010 and February 2021 (Table 1). Magnetic resonance imaging scanning of all data sets was performed on the same 1.5 Tesla Magnetom Symphony (Siemens Medical, Erlangen, Germany); the study protocol consisted of a T1w scan with 144 slices [256 × 256 pixels, slice thickness 1.2 mm, pixel size 1.0 × 1.0 mm, echo time (TE) 4.2 ms, repetition time (TR) 1,640 ms] and a DTI study protocol with 52 volumes (64 slices, 128 × 128 pixels, slice thickness 2.8 mm, pixel size 2.0 × 2.0 mm, 39 control subjects, 306 ALS subjects), 48 gradient directions (*b* = 1,000 s/mm^2^), and four scans with *b* = 0, TE = 95 ms, TR = 8,000 ms (DTI protocol A). Alternatively, DTI protocol B consisted of 39 gradients including three b0 gradient directions (*b* = 1,000 s/mm^2^, voxel size 2.0 × 2.0 × 2.8 mm, 128 × 128 × 64 matrix, TE = 95 ms, TR = 8,000 s, 0 control subjects, 33 ALS subjects); DTI protocol C consisted of 2 × 31 gradient directions including two b0 gradient directions (*b* = 1,000 s/mm^2^, voxel size 2.5 × 2.5 × 2.5 mm, 64 slices, 128 ×128 in-plane matrix, TE = 102 ms, TR = 8,700 ms, 59 control subjects, 62 ALS subjects). The study was performed according to institutional guidelines in accordance with the Declaration of Helsinki and was approved by the Ethical Committee of the University of Ulm (reference # 19/12).

**Table 1:** Demographic and clinical features of the subjects.

	**Control subjects (***n*** = 98)**	**ALS subjects (*****n*** **= 404)**			
Age in years	56.75 ± 15.91	63.05 ± 11.48			*p* <0.001
Gender	*w* = 49, *m* = 49	*w* = 167, *m* = 237			n.s.
		**Training**	**Test**		
		**(***n*** = 98)**	**(***n*** = 306)**		
Disease duration in months		19.25 ± 17.63	18.66 ± 7.16	n.s.	
ALS-FRS		39.04 ± 22.81	39.64 ± 6.98	n.s.	

*Values are mean ± SD. Significance was tested using the t-test for age, disease duration, and ALS-FRS, and the Fisher exact test for gender. ALS-FRS, ALS functional rating scale (11). n.s., not significant (p > 0.05)*.

The software *Tensor Imaging and Fiber Tracking* (TIFT) was used for data analysis (12). Prior to data analysis, data underwent a standardized quality control. The DTI data were stereotaxically normalized to the Montreal Neurological Institute (MNI) stereotaxic space using a group-specific template (13); in a consecutive step, fractional anisotropy (FA) maps were corrected for the covariate age (14). For the harmonization of FA differences resulting from different acquisition protocols, FA maps were harmonized according to a previously reported protocol by a linear first order correction (15) and have been successfully applied to DTI data even from different scanners (16, 17). Finally, an 8 mm (FWHM) Gaussian filter was applied for smoothing of FA maps in order to achieve a good balance between sensitivity and specificity (13). For tractwise fractional anisotropy statistics (TFAS), the following tracts of interest (TOI) were isolated (18): the CST, the corticopontine tract, the corticorubral tract, the corticostriatal pathway, the proximal portion of the perforant path, and tracts originating in segments II and III of the corpus callosum (19–21). Using a threshold of 0.2, the mean FA-value within each TOI was calculated (22). The texture data calculation of the corpus callosum was described in detail in a previous study (10). In brief, after alignment to the AC-PC line (anterior and posterior commissure) and rigid brain transformation, the corpus callosum was automatically segmented from median and paramedian sagittal T1w slices. For the segment III, the texture was analyzed and several texture parameters were calculated, notably texture homogeneity and entropy, each age-corrected. The texture and the FA data were registered for consecutive machine learning operations.

### Machine Learning Classifiers

According to previous studies, the following variables were defined as features in the applied machine learning classifiers (10, 19): age-corrected mean FA of the CST, the corticopontine tract, the corticorubral tract, the corticostriatal pathway, the proximal portion of the perforant path, and tracts originating in the segments II and III of the corpus callosum, as well as age-corrected texture homogeneity and entropy from segment III of the corpus callosum. Age-correction was important, due to significant differences between groups (see Table 1). For the diffusion metrics, FA was exclusively chosen out of the DTI metrics, since it has been identified as the most robust DTI-based parameter, according to a recent systematic review (7). All parameters were z-transformed prior to further analyses. For the dichotomous classification ALS patients vs. controls, two output classes were defined.

Two machine learning classification models were employed, i.e., first, a linear support vector machine (SVM) (23), which is a very robust model that also allowed us to analyze the linear coefficients of the model for later interpretation and second, a multilayer perceptron (MLP) classifier (24) in order to verify the results from the SVM. Each hyperparameter was chosen *a priori*. In the majority of the hyperparameters, this was done by using default values or referring to prior work (24). Choosing the hyperparameters *a priori* was a design choice, as we focused on the integration of multiparametric MRI data, not on optimizing or comparing ML models. This approach also had the consequence that the hyperparameters were not fitted to the validation set, except for the hidden layer sizes in the MLP. Calculations were done using the scikit-learn 0.23.2 library for python (25). To assess the diagnostic power of our models, receiver operating characteristics (ROC) curves were used to calculate the area under the curve (AUC) (26, 27).

### Linear Support Vector Machine

For the SVM, we decided to use the “svc” estimator from the “svm” class with the following (hyper) parameters: *kernel* = “*linear”* for a linear SVM, *class_weight* = “*balanced”* to account for the sample size imbalance, and *max_iter* = −1 to ensure convergence during training (19, 21). For all other parameters, the default value was used, including the regularization parameter *C* = 1. The class weights were implemented as coefficients to the regularization parameter *C* for each class. This way, inaccurate classification for the smaller control group were penalized more heavily than for the larger ALS group, addressing the issue of imbalanced class sizes. By implementing class weights, we favored the benefit of having more examples over the downside of class imbalance, in the case of the SVM. The model was fit using all 404 ALS subjects and all 98 control subjects. For validation, a leave-one-out cross-validation (LOOCV) was performed (23, 25). For the interpretation of the input parameter weights, the “*coef_”* attribute was called (25). The entire procedure was repeated for each MRI modality, effectively resulting in three models, i.e., one with all features, one with only FA data, and one with only texture data.

### Multilayer Perceptron

**Figure 1:**
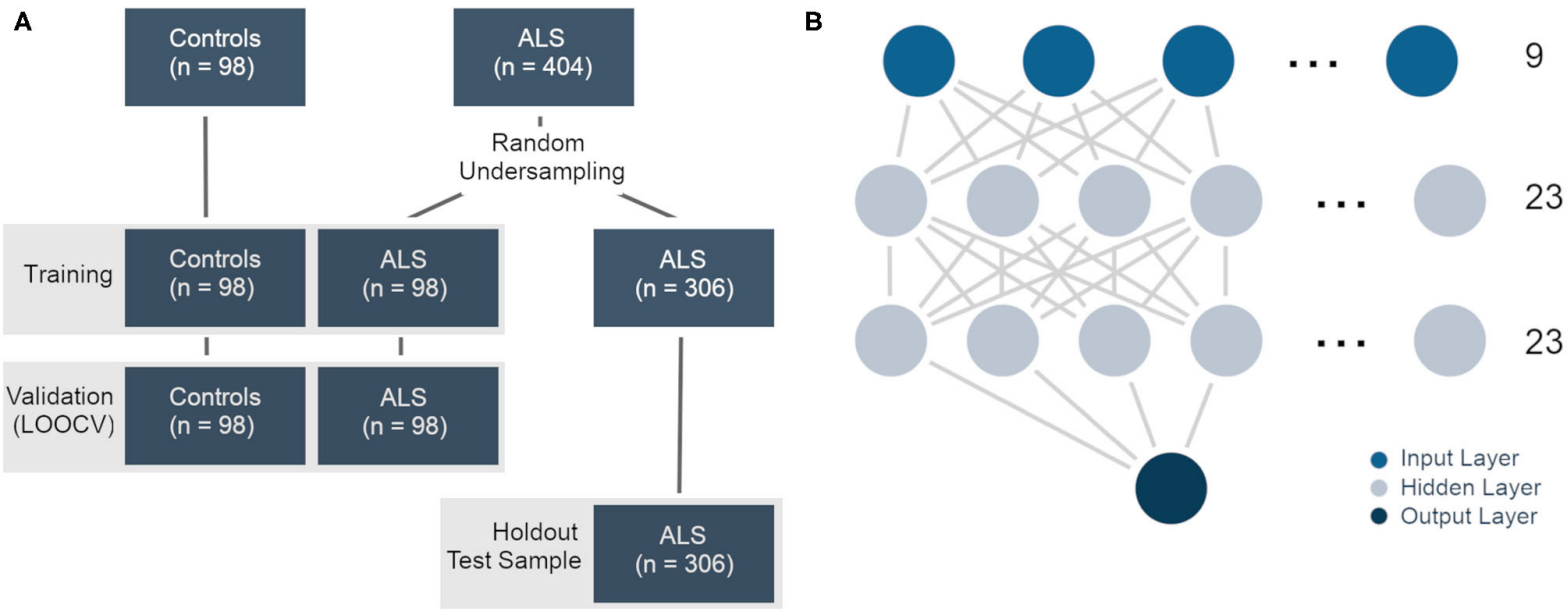
**(A)** Training, validation, and test samples in the multilayer perceptron classifier. The ALS data were undersampled for the training and validation samples. The leftover data were used as a holdout test sample. **(B)** Graphical representation of the multilayer perceptron/neural network. There were nine features in the input layer and two classes in the output layer (ALS, controls). The 23 nodes in the two hidden layers were determined by an extensive grid search.

To verify the results from the SVM classifier, the MLPClassifier estimator from the “neural-network” class was chosen. We modeled this estimator in close resemblance to a previous model by van der Burgh et al. (24), using the following hyperparameters: *activation* = “*logistic”* for a logistic unit activation function, *alpha* = 0.1 to set the L2 penalty parameter to 0.1 in order to prevent overfitting, and *max_iter* = 1,000 to ensure convergence during training. The hidden layer sizes [*hidden_layer_sizes* = (23,23)] were determined by an exhaustive grid search within a 1–500 interval. For all other parameters, the default value was used. Because the MLPClassifier struggles with unbalanced sample sizes, we undersampled the ALS subject group at random to match the control group in size (*n* = 98). The model was then fit using these 98 ALS subjects and all 98 control subjects. For validation, a LOOCV was performed. Finally, the model was tested on the ALS patients' data that had been excluded during the undersampling process (*n* = 306, referred to as “holdout test sample”) (see Figure 1).

## Results

### Linear Support Vector Machine

**Figure 2:**
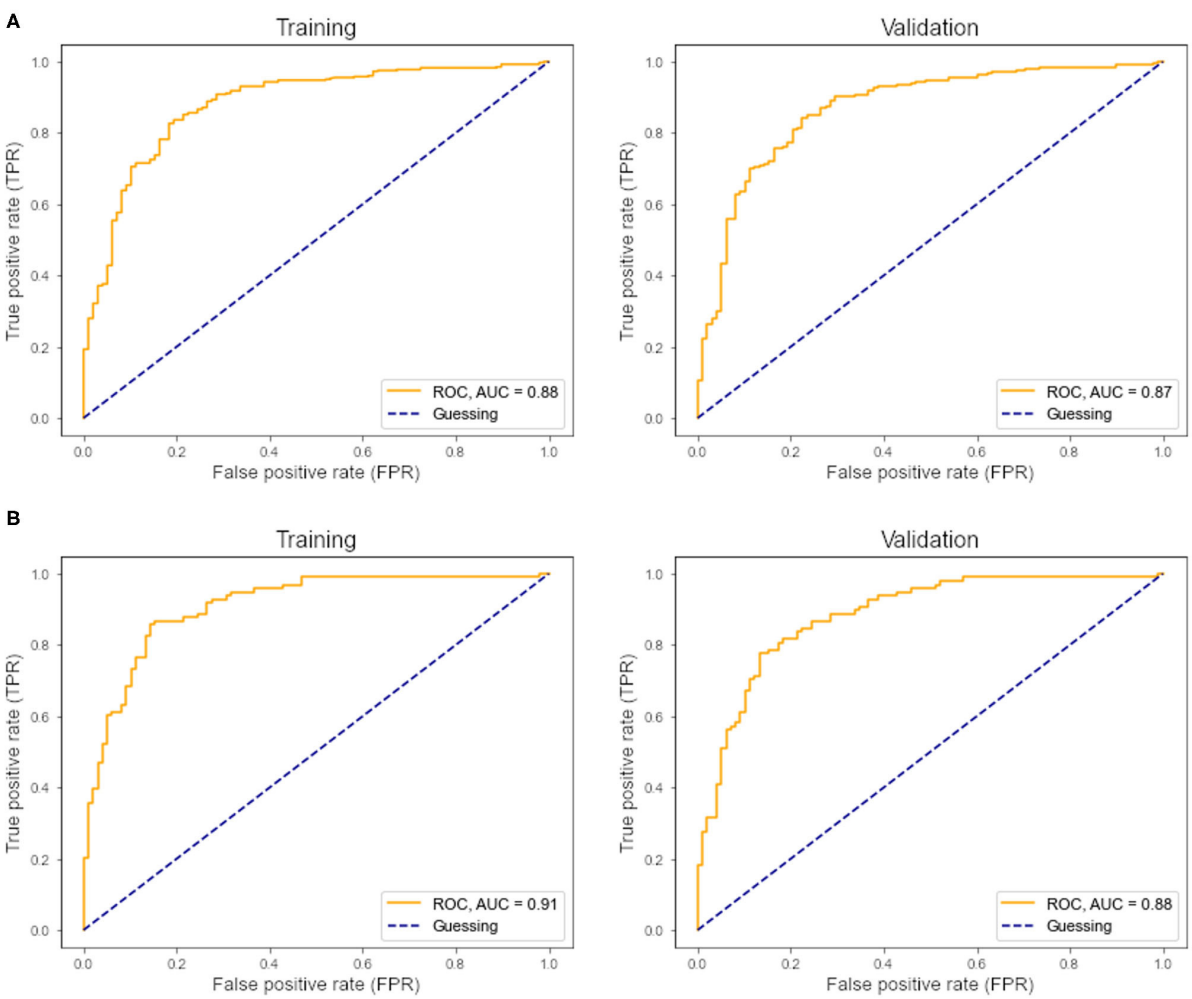
**(A)** ROC curve and AUC from the support vector classifier. Left, training sample; right, leave-one-out cross-validation. **(B)** ROC curve and AUC from the multilayer perceptron classifier. Left, training sample; right, leave-one-out cross-validation.

Within the training sample, the linear SVC achieved 81% sensitivity and 82% specificity with an AUC of 88% in the ROC analysis (Figure 2A). The LOOCV analysis confirmed these results with 80% sensitivity, 80% specificity, and an AUC of 87% in the ROC analysis. Each modality (FA maps, texture data) on its own underperformed the combined model (Table 2). Analysis of the (linear) coefficients demonstrated that the corpus callosum texture homogeneity contributed the most to accurate classification, followed by the CST FA and corpus callosum FA (Table 3).

**Table 2:** Sensitivity and specificity of the linear support vector classifier.

	**Specificity (%)**	**Sensitivity (%)**	**Youden-index (%)**	**AUC**
**Training**				
All data	82	81	63	0.88
FA data	72	65	38	0.76
Texture data	77	76	53	0.85
**Validation**				
All data	80	80	60	0.87
FA data	67	64	31	0.72
Texture data	77	76	53	0.84

*AUC, area under curve in receiver operating characteristics curve*.

**Table 3:** Linear coefficients of the support vector classifiers.

	**CC segment III homogeneity**	**CC segment III entropy**	**CST FA**	**Corticopontine tract FA**	**Corticorubral tract FA**	**Corticostriatal pathway FA**	**Proximal perforant path FA**	**CC segment II FA**	**CC segment III FA**
All data	1.08	−0.06	−0.53	−0.13	−0.04	0.35	−0.16	−0.37	0.46
FA	–	–	−0.68	−0.40	−0.06	0.82	−0.13	−0.22	−0.26
Texture	1.00	−0.11	–	–	–	–	–	–	–

*CC, corpus callosum; CST, corticospinal tract; FA, fractional anisotropy*.

### Multilayer Perceptron

Within the training sample, the MLP classifier achieved 81% sensitivity and 86% specificity with an AUC of 0.91 (“excellent”) in the ROC analysis (Figure 2B). The LOOCV confirmed these results with 79% sensitivity, 84% specificity, and an AUC of 0.88 (“good”) in the ROC analysis. In the holdout test-sample (Figure 1A), the MLP classifier reached 72% sensitivity.

## Discussion

The present study demonstrated the potential of a multiparametric approach in the diagnostic classification of ALS, as a proof of concept.

### Classifiers

The SVM classifier delivered the most robust results with 80–81% sensitivity, 80–82% specificity, and an AUC of 0.87–0.88 (“good”) in the ROC analysis. Analyses of the coefficients revealed that texture homogeneity of the callosal area III was most important in distinguishing ALS from controls, followed by CST FA and corpus callosum FA. The high diagnostic accuracy of texture data alone was surprising, as we expected diffusion metrics to be superior in describing neurodegeneration in ALS, especially in the CST, as demonstrated in a study using a Random Forest approach to assess the clinical utility of DTI by combining FA, mean diffusivity (MD), and radial diffusivity (RD) measured along tracts between the cerebral peduncle and the corona radiata, reaching a mean five-fold cross validation accuracy of 80% in discriminating ALS from controls (28). Yet, prior studies reported similar results using texture (10) and DTI data (29). In addition, we noticed that the stage-defining tracts besides the CST did not contribute much to the classification, especially when texture data were present. Since adding a feature whose information is already (fully) represented by another does not increase accuracy, some DTI information might be redundant in the use of diffusion data of the CST or the corpus callosum or texture data. In a previous approach, Fekete and co-workers have addressed multicollinearity by using a multiple kernel learning (MKL) approach and implementing a nifty optimization method for the weights of the kernel matrix sum (30). Also, late-stage tracts will most probably only be affected in a subset of ALS patients—this might be why these data did not distinguish well-enough between patients and controls in our model (31). Pruning these features in future models might increase performance, as the subject to feature ratio increases without necessarily losing predictive power. The neural network classifier achieved higher performance metrics compared to the SVM, with an AUC of 0.87–0.91 (“good” to “excellent”) in the ROC curve. However, only 72% sensitivity was reached in the holdout test-sample. This result is probably due to overfitting, as well as some misrepresentation of the cohort by the random undersampling of ALS subjects. If the model overfits the training sample or the test sample differs substantially from the training sample, loss of predictive performance is expected. Still, we consider these results to be a proof of concept of what can be achieved with sound feature selection and a reasonable amount of multiparametric data.

The present study used only microstructural data, in a line of agreement with a previous study that reached a mean five-fold cross validation accuracy of 80% in discriminating ALS from controls from tract-based DTI metrics (28). Future studies should extend on this by incorporating additional MRI parameters like gray matter volume and functional connectivity. For functional connectivity data, machine learning has been already applied to independent component analysis, demonstrating an accuracy of 71.5% (4). The combination of different models, for example in a stacking ensemble machine learning classification, might further increase diagnostic accuracy (9). In a recent study on longitudinal neurodegeneration in the brain in ALS, data from different modalities were combined using deep learning (32). In this study, a random walker model did not only predict disease propagation in ALS, but also contributed to correct survival prediction. Applying deep learning techniques to disease classification might increase diagnostic accuracy in future models.

### Limitations

Although the number of subjects with ALS was rather high, the machine learning models were trained with a comparatively low number of control subjects. This sample size hampered our model's performance in two respects, first by the limited data quantity of *n* <100 control samples and second by class imbalance. In addition, age differed significantly between ALS patients and controls; however, data analysis included age correction in order to compensate for this difference. Given that this is a proof-of-concept study, we advise against using this machine learning model in a clinical setting, as the field is still developing and scientific consensus regarding diagnostic utility of machine learning in ALS has not been reached yet. In addition, a proper clinical classifier has to incorporate mimic disorders, as only these are (falsely) suspected to be ALS cases. It can be argued that many ALS mimics are peripheral neuropathies with normal cranial MRI, hence, training a model with healthy control data and fine-tuning it with mimic disorders might suffice.

### Conclusion

The integration of multimodal microstructural neuroimaging data into an appropriate diagnostic classifier demonstrated the power of machine learning for multiparametric, quantitative neuroimaging, and this proof of concept allowed for an accurate dichotomous classification of ALS patients vs. controls. Extending this concept beyond microstructural data might further enhance diagnostic accuracy in future models.

## Data Availability Statement

The raw data supporting the conclusions of this article will be made available by the authors, without undue reservation.

## Ethics Statement

The study was performed according to institutional guidelines in accordance with the Declaration of Helsinki and was approved by the Ethical Committee of the University of Ulm (reference # 19/12). The patients/participants provided their written informed consent to participate in this study.

## Author Contributions

TK: study concept and design, analyses and interpretation of data and drafting of manuscript. JK: study concept and design, interpretation of data, and critical revision of manuscript for intellectual content. H-PM: study concept and design, analyses and interpretation of data, and critical revision of manuscript for intellectual content. AL and AB: interpretation of data and critical revision of manuscript for intellectual content. All authors contributed to the article and approved the submitted version.

## Conflict of Interest

The authors declare that the research was conducted in the absence of any commercial or financial relationships that could be construed as a potential conflict of interest.

## Publisher's Note

All claims expressed in this article are solely those of the authors and do not necessarily represent those of their affiliated organizations, or those of the publisher, the editors and the reviewers. Any product that may be evaluated in this article, or claim that may be made by its manufacturer, is not guaranteed or endorsed by the publisher.
